# The causal relationship between multiple autoimmune diseases and nasal polyps

**DOI:** 10.3389/fimmu.2023.1228226

**Published:** 2023-08-24

**Authors:** Siyuan Chen, Lu Tan, Danxue Qin, Hao Lv, Kunyu Liu, Yingying Xu, Xiaomin Wu, Jingyu Huang, Yu Xu

**Affiliations:** ^1^ Department of Otolaryngology-Head and Neck Surgery, Renmin Hospital of Wuhan University, Wuhan, China; ^2^ Department of Rhinology and Allergy, Renmin Hospital of Wuhan University, Wuhan, China; ^3^ Research Institute of Otolaryngology-Head and Neck Surgery, Renmin Hospital of Wuhan University, Wuhan, China; ^4^ Hubei Province Key Laboratory of Allergy and Immunology, Wuhan University, Wuhan, China

**Keywords:** autoimmune diseases, nasal polyps, two-sample Mendelian randomization, rheumatoid arthritis, adult-onset Still’s disease

## Abstract

**Background:**

Although previous sporadic studies have reported the associations between a few autoimmune diseases and nasal polyps, these studies have limitations such as conflicting results, small sample sizes, and low levels of evidence.

**Methods:**

Several autoimmune diseases were selected as exposures while the nasal polyps were selected as outcomes. Bidirectional univariable Mendelian randomization and multivariable Mendelian randomization analyses were performed after rigorous screening of instrumental variables. Then mediation analyses were conducted to further investigate the underlying mechanisms.

**Results:**

For the first time, we investigated the causal relationships between nine autoimmune diseases and nasal polyps in different genders and found: (1) there was a causal association between adult-onset Still's disease and nasal polyps; (2) sarcoidosis, ulcerative colitis, type 1 diabetes, and Crohn’s disease had no significant associations with nasal polyps; (3) celiac disease showed a suggestive positive association with female nasal polyps, whereas juvenile arthritis and multiple sclerosis showed suggestive positive associations with male nasal polyps. By contrast, arthropathic psoriasis showed a suggestive negative association with nasal polyps. In addition to these nine diseases, previous controversial issues were further investigated: (1) there was a causal relationship between rheumatoid arthritis and nasal polyps, which was partially mediated by “BAFF-R for IgD+ B cells”; (2) ankylosing spondylitis showed suggestive positive associations with the female but not the male nasal polyps. Besides, we validated that there was no causal effect of autoimmune hyperthyroidism on nasal polyps.

**Conclusion:**

Specific conclusions regarding the causal effects of multiple autoimmune diseases on nasal polyps are the same as above. By comparing results between different genders, we have initially observed the sex bimodality in the causal effects between autoimmune diseases and nasal polyps, with those on male nasal polyps being stronger than those on female nasal polyps. Our study lays a solid foundation for further research in the future, not only helping identify individuals susceptible to nasal polyps early but also improving our understanding of the immunopathogenesis of these heterogeneous diseases.

## 1 Introduction

Chronic rhinosinusitis (CRS), which can be subdivided into chronic rhinosinusitis without nasal polyps (CRSsNP) and chronic rhinosinusitis with nasal polyps (CRSwNP), is a multifactorial and heterogeneous disease characterized by swelling, fluid accumulation, and fibrin deposition. Nasal polyps (NPs), the most common comorbidity of CRS, can obstruct the sinus ostium and nasal passage, leading to the symptoms of nasal obstruction, rhinorrhea, hyposmia, and sinus pressure (1, 2). The refractory nature of NPs imposes a heavy healthy, psychological, and financial burden worldwide, highlighting the urgency of further research into the causes and pathogenesis of NPs.

Previous studies of molecular biological and immunological experiments have shown increased expression of anti-dsDNA antibodies in nasal polyp tissue, but not in the uncinate process and inferior turbinate (3). Furthermore, there was no elevated anti-dsDNA in the tissues of patients with CRSsNP (4), indicating that chronic inflammation per se does not increase autoantibody levels. These findings suggest that multiple autoimmune diseases may be associated with the development of NPs. Accordingly, although exceedingly rare, several studies have explored the association between a few autoimmune diseases (ADs) and chronic rhinosinusitis (CRS). Some parts of these studies have yielded consistent results, such as both findings of Shih et al. (5) and Tan et al. (6) reported that systemic lupus erythematosus is not significantly associated with NPs. However, more often, their results are conflicting, such as the results in exploring the association between ankylosing spondylitis and NPs (5, 7), as well as the association between rheumatoid arthritis with NPs (5–7). These conflicts are largely attributed to problems such as relatively small sample sizes and susceptibility to selection bias. Moreover, previous studies also have limitations such as lack of causality and limited coverage of autoimmune diseases. These issues hinder the exploration process of the relationship between autoimmune diseases and NPs, indicating the necessity of finding more ideal methods for research.

Mendel’s second law suggests that alleles for traits are passed on independently of each other, i.e., the selection of one trait’s allele is unrelated to the selection of another trait’s allele. As a result, the genetic variations are distinct from self-chosen actions and are determined long before the disease’s beginning, which minimizes the problems of reverse causality and confounding (8). Two-sample Mendelian randomization (MR) based on large-scale genome-wide association studies (GWAS), using single-nucleotide polymorphisms (SNP) of various traits as instrumental variables (IV) to study the association between exposure trait and outcome trait, has advantages such as large sample size and high level of evidence. Therefore, we applied two-sample bidirectional MR analyses to comprehensively assess the potential causal relationship between ADs and NPs, while attempting to reveal the underlying mechanisms between them.

## 2 Methods

### 2.1 Data source

#### 2.1.1 Autoimmune diseases

To assess the causal relationship more comprehensively between multiple ADs and nasal polyps, the following traits were selected by reviewing previous studies and searching various GWAS databases. ① Connective tissue diseases (adult-onset Still’s disease (AOSD), sarcoidosis, juvenile arthritis, ankylosing spondylitis, rheumatoid arthritis (RA), arthropathic psoriasis). ② Neuromuscular disease (multiple sclerosis). ③ Endocrine system diseases (type 1 diabetes, autoimmune hyperthyroidism). ④ Digestive system diseases (ulcerative colitis, Crohn’s disease, celiac disease). Details are available on the website of *“Frontiers in Immunology”* (**Table S1**). All data were approved by the ethical review boards to which the original investigators belonged, and each participant in the original study provided informed consent to the corresponding original investigators.

#### 2.1.2 Nasal polyps

Several clinical studies of nasal polyps have found that the incidence and postoperative recurrence rates of CRSwNP were higher in men than in women (2, 6, 9), while female patients had higher symptom scores than male patients (10), suggesting potential heterogeneity between CRSwNP patients of different genders. Therefore, summary data on gender-specific nasal polyps were obtained from the UK Biobank (UKB) consortium. The data did not distinguish between subtypes such as eosinophilic or non-eosinophilic polyps, or whether surgical treatment was received. Details are available on the website of *“Frontiers in Immunology”* (**Table S1**). All data were approved by the ethical review boards to which the original investigators belonged, and each participant in the original study provided informed consent to the corresponding original investigators.

#### 2.1.3 IV selection

**Figure 1:**
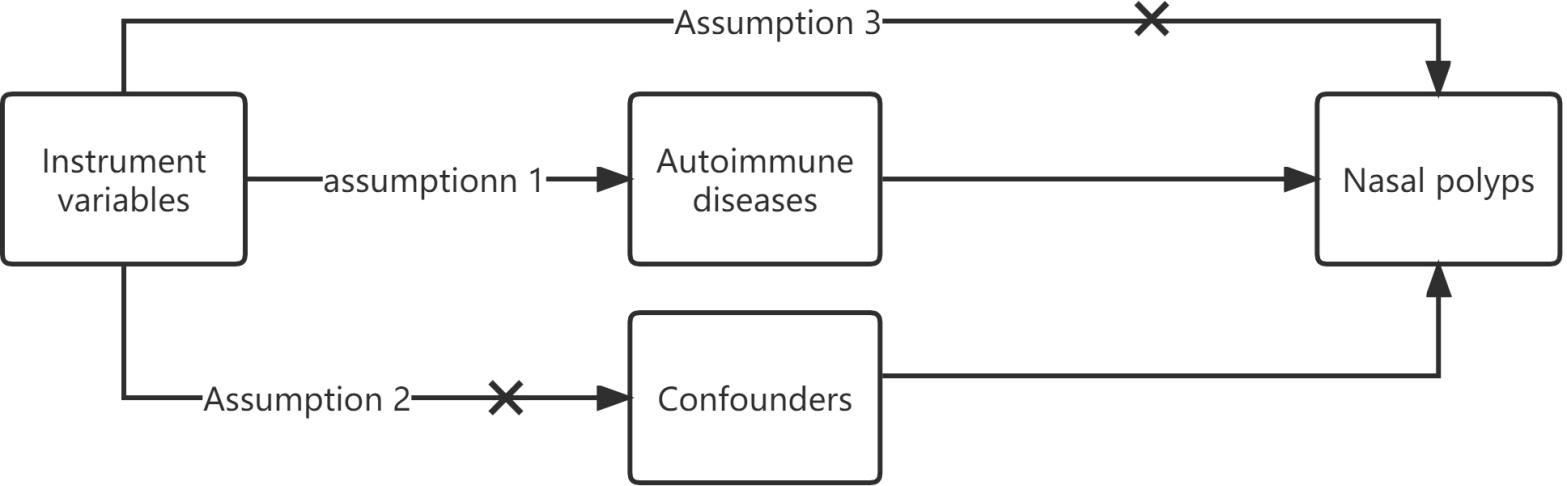
The three fundamental assumptions of MR instrument variables.

The three fundamental assumptions of MR are as follows: 1. The instrument variable (IV) is related to the risk factor. 2. The IV is unaffected by confounding variables between exposure and outcome. 3. The IV has no direct effect on the outcome but only influences it through exposure (**Figure 1**) (11, 12).

The “TwoSampleMR” package was used to clump identified SNPs with genome-wide significance (*p* < 5E−8) for independence (13). Clumping was performed with a strict cut-off of *R^2^* < 0.001 and a window of 10,000 kb, the 1000 Genomes European data were used as the reference panel. In cases where SNPs were in linkage disequilibrium, those with the lowest *p*-value were retained. No proxy SNPs were used in our study. To control for potential confounding, we examined each instrumental SNP in the PhenoScanner GWAS database (http://www.phenoscanner.medschl.com.ac.uk/) to assess any prior associations (*p* < 5E-8) with plausible confounders (14, 15).

To meet the requirement that IVs should only be associated with the outcome through exposure, we excluded SNPs that were strongly associated with the outcome (*p* < 5E-8). The effects of the SNPs on exposure and outcome were then harmonized to ensure that the effect estimates corresponded to the same allele. Finally, the *F* statistic was used to measure the strength of the IVs. Weak IVs, defined as having an *F* statistic less than 10, were excluded to avoid bias (16).

### 2.2 MR analysis

#### 2.2.1 Univariable MR

**Figure 2:**
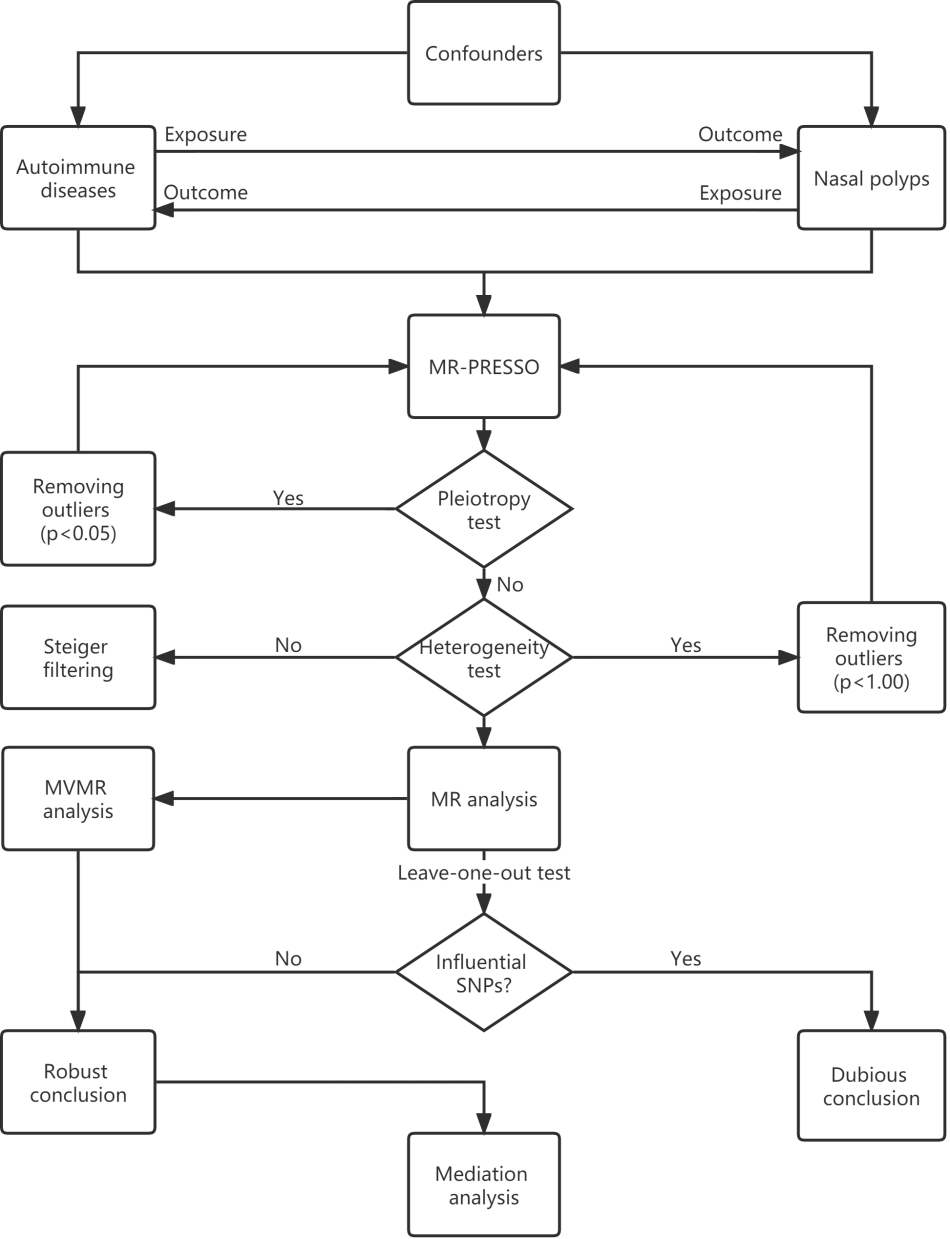
MR flowchart.

Univariable MR is the fundamental method for exploring the causal effect of an exposure trait on an outcome trait. As shown in the flowchart (**Figure 2**), the MR-PRESSO global test was used to evaluate horizontal pleiotropy and to remove outliers. The inverse variance weighting (IVW) method was then used to assess the heterogeneity of the SNPs retained after pleiotropy correction. The Cochran’s *Q* statistic was used to detect the presence of heterogeneity, and the SNPs with *p* < 1.00 were removed from the MR-PRESSO analysis if heterogeneity was significant (*p*-value of Cochran’s *Q* statistic < 0.05). We also performed the MR Steiger test to estimate the potential reverse causal impact of ADs on NPs.

MR analyses were conducted using the IVW method. As the IVW method ignores the invalid IVs and pleiotropic effects, we also performed sensitivity analyses using the weighted median, MR-Egger regression, weighted model, and “leave-one-out” analysis. The weighted median method requires valid SNPs to account for at least 50% of the weights in the meta-analysis (17), the MR-Egger regression is based on the assumption of instrument strength independent of direct effect (InSIDE), if the InSIDE hypothesis is satisfied, MR-Egger regression can evaluate the existence of pleiotropy with the intercept term (18). By contrast, if the InSIDE assumption is violated, the weighted model has greater power to detect a causal effect with less bias, and lower type I error rates than the MR-Egger regression does (19). The “leave-one-out” analysis could identify certain extreme SNPs.

The ORs and their 95% confidence intervals (95% CI) are used to present the results. The possibility of false-positive results from multiple hypothesis testing requires adjustment of the *p*-value for correction. As the Bonferroni method is too conservative, the Benjamini-Hochberg method was used for correction. Associations with *FDR* < 0.05 were considered significant, while associations with *FDR* > 0.05 and *p*-values < 0.05 were considered suggestive. All analyses were performed using “TwoSampleMR”, “MendelianRandomization”, “MRPRESSO”, and other necessary R packages in version 4.2.2 of R.

#### 2.2.2 Multivariable MR

Multivariable MR (MVMR) can estimate the direct causal effects of exposure traits that have confounding factors among each other. Therefore, we conducted multivariable MR analyses to estimate the direct causal effects of each autoimmune disease (having shared IVs among several exposure traits) on the nasal polyps of females and males. The *OR*s and their 95% confidence intervals are used to present the results. A *p*-value < 0.05 was considered statistically significant. All analyses were conducted using “TwoSampleMR”, “MendelianRandomization”, “MRPRESSO”, and other necessary R packages in version 4.2.2 of R.

#### 2.2.3 Mediation analysis

Mediation analysis explores the mediation traits that mediate the causal relationship between the exposure trait and the outcome trait. To identify mediators between significant MR associations, two-step mediation analyses were conducted. First, genetic IVs for significant exposure were used to assess the causal effect of exposure on potential mediators. Second, genetic IVs for identified mediators were used to assess the causal effect of the potential mediator on nasal polyps. The product of the coefficients approach was used to estimate the indirect effect of exposures on nasal polyps via the potential mediator. The delta method was used to derive standard errors for the indirect effects.

## 3 Results

We obtained the instrumental variables for each exposure respectively. After implementing quality control measures, the mean *F* statistic for every instrument variable-exposure association was greater than 10, indicating a low possibility of weak instrumental variable bias (**Tables S2-3**). The final MR-PRESSO global test for each exposure trait did not detect any significant horizontal pleiotropy, while the Cochran’s *Q* test and the MR-Egger regression showed no significant evidence of heterogeneity and pleiotropy (**Tables S4-5**), suggesting that the IVs included in the MR analysis are valid. Moreover, the MR Steiger test showed no evidence of reverse causality in any of the analyses (**Tables S4-5**). Details are available on the website of *“Frontiers in Immunology”.*

### 3.1 Univariable MR results

#### 3.1.1 Univariable MR results for female

**Figure 3:**
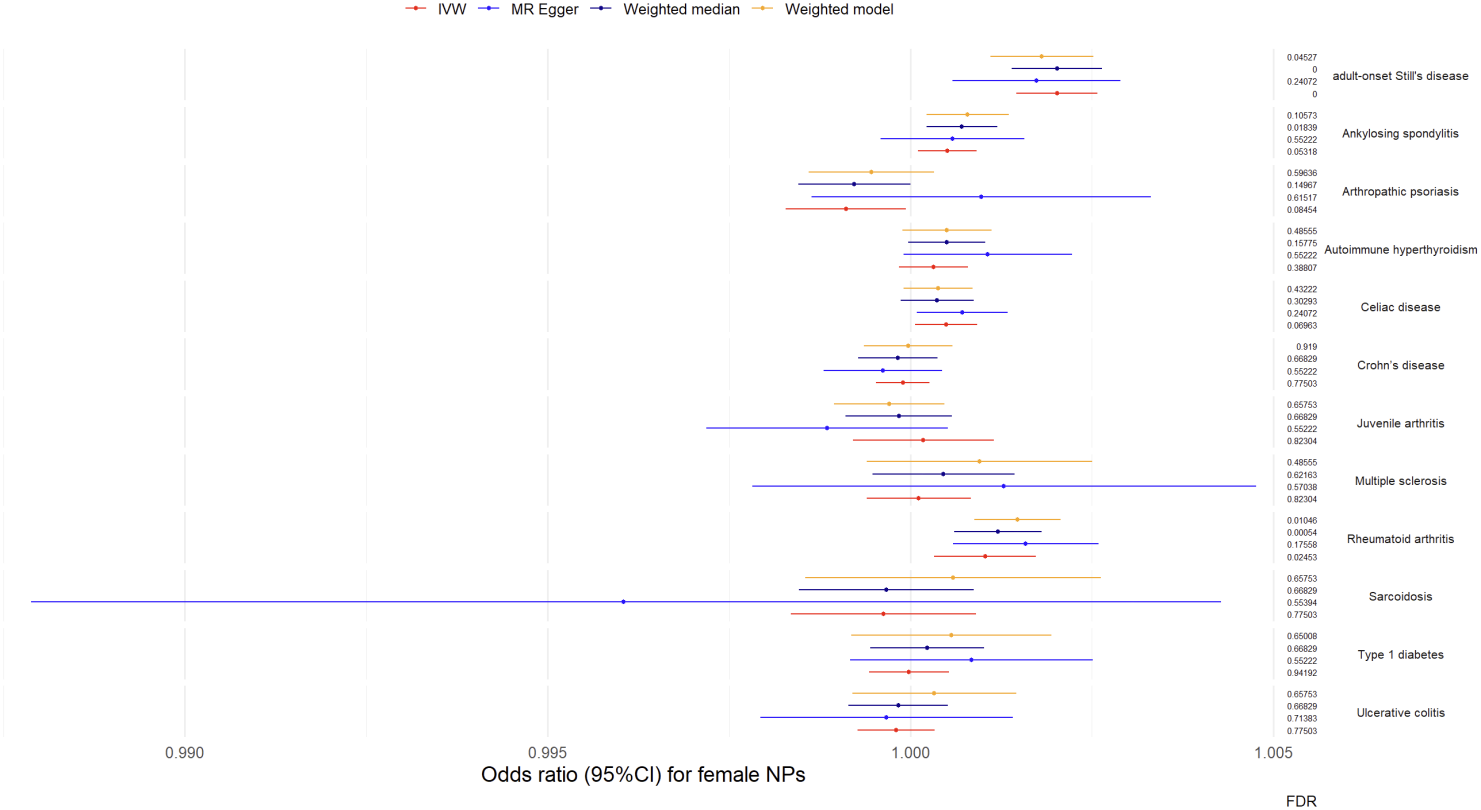
Odds ratio (95%*CI*) for female nasal polyps.

Results from univariable MR analyses showed causal associations between multiple ADs and NPs in females (**Figure 3**, **Table 1**). MR estimates from the IVW method suggested that one standard deviation (1-*SD*) increase of inverse-normal transformed risk of AOSD (*OR* = 1.002018, 95% *CI* 1.001461-1.002576), rheumatoid arthritis (*OR* = 1.001027, 95% *CI* 1.000326–1.001729) showed a significant positive association with female NPs risk, whereas 1-*SD* increase of inverse-normal transformed risk of autoimmune hyperthyroidism (*OR* = 1.000317, 95% *CI* 0.999839–1.000794), Crohn’s disease (*OR* = 0.999896, 95% *CI* 0.999527–1.000265), juvenile arthritis (*OR* = 1.000177, 95% *CI* 0.999206–1.001149), multiple sclerosis (*OR* = 1.000114, 95% *CI* 0.999398–1.000832), sarcoidosis (*OR* = 0.999627, 95% *CI* 0.998351–1.000904), type 1 diabetes (*OR* = 0.999979, 95% *CI* 0.999427–1.000532), and ulcerative colitis (*OR* = 0.9998, 95% *CI* 0.99927–1.000331) were not significantly associated with female NPs risk. Of note, 1-*SD* increase of inverse-normal transformed risk of ankylosing spondylitis (*OR* = 1.000509, 95% *CI* 1.000106–1.000912) and celiac disease (*OR* = 1.000494, 95% *CI* 1.000067–1.000922) showed a suggestive positive association with female NPs risk, whereas the risk of arthropathic psoriasis (*OR* = 0.99911, 95% *CI* 0.998282–0.999938) showed a suggestive negative association with female NPs risk.

**Table 1:** MR results for female.

traits	method	*OR*	*95% CI*	*p*	*FDR*
Adult-onset Still’s disease	IVW	1.002018	1.001461-1.002576	1.22E-12	1.46E-11
	MR Egger	1.001738	1.000582-1.002896	0.060179	0.240716
	Weighted median	1.002019	1.001395-1.002643	2.14E-10	2.57E-09
	Weighted model	1.00181	1.001098-1.002521	0.007545	0.045268
Ankylosing spondylitis	IVW	1.000509	1.000106-1.000912	0.013294	0.073117
	MR Egger	1.000579	0.999587-1.001572	0.290051	0.571248
	Weighted median	1.000709	1.000219-1.0012	0.004597	0.025285
	Weighted model	1.000787	1.000219-1.001355	0.026433	0.145381
Arthropathic psoriasis	IVW	0.99911	0.998282-0.999938	0.035224	0.096867
	MR Egger	1.000975	0.998638-1.003317	0.563906	0.620297
	Weighted median	0.999225	0.998451-0.9999996	0.049888	0.180752
	Weighted model	0.999463	0.998598-1.000328	0.347879	0.637778
Autoimmune hyperthyroidism	IVW	1.000317	0.999839-1.000794	0.194034	0.426874
	MR Egger	1.001066	0.999904-1.002229	0.214115	0.571248
	Weighted median	1.000499	0.999968-1.001031	0.065728	0.180752
	Weighted model	1.000502	0.999890-1.001114	0.206345	0.534106
Celiac disease	IVW	1.000494	1.000067-1.000922	0.023211	0.085107
	MR Egger	1.000715	1.000091-1.00134	0.046266	0.254461
	Weighted median	1.000368	0.999865-1.000872	0.151467	0.333227
	Weighted model	1.000381	0.999903-1.00086	0.144073	0.528268
Crohn’s disease	IVW	0.999896	0.999527-1.000265	0.581269	0.799245
	MR Egger	0.999621	0.998807-1.000436	0.368146	0.571248
	Weighted median	0.999826	0.99928-1.000372	0.531162	0.668293
	Weighted model	0.999968	0.999359-1.000578	0.919	0.919
Juvenile arthritis	IVW	1.000177	0.999206-1.001149	0.720865	0.829896
	MR Egger	0.998851	0.997188-1.000517	0.308899	0.571248
	Weighted median	0.99984	0.999108-1.000573	0.668293	0.668293
	Weighted model	0.999705	0.998945-1.000465	0.501978	0.663006
Multiple sclerosis	IVW	1.000114	0.999398-1.000832	0.754451	0.829896
	MR Egger	1.001286	0.997823-1.004761	0.475316	0.580941
	Weighted median	1.000456	0.999475-1.001437	0.362615	0.664794
	Weighted model	1.000951	0.999399-1.002505	0.242776	0.534106
Rheumatoid arthritis	IVW	1.001027	1.000326-1.001729	0.004088	0.044970
	MR Egger	1.001588	1.000584-1.002593	0.014631	0.160946
	Weighted median	1.001207	1.000603-1.001811	8.93E-05	0.000982
	Weighted model	1.001478	1.000884-1.002072	0.000872	0.00959
Sarcoidosis	IVW	0.999627	0.998351-1.000904	0.566727	0.799245
	MR Egger	0.996049	0.987882-1.004283	0.415453	0.571248
	Weighted median	0.999666	0.998462-1.000871	0.586598	0.668293
	Weighted model	1.000586	0.998552-1.002625	0.602733	0.663006
Type 1 diabetes	IVW	0.999979	0.999427-1.000532	0.941924	0.941924
	MR Egger	1.000839	0.999167-1.002513	0.339262	0.571248
	Weighted median	1.00023	0.999446-1.001015	0.56488	0.668293
	Weighted model	1.000563	0.999186-1.001942	0.433389	0.663006
Ulcerative colitis	IVW	0.9998	0.99927-1.000331	0.460155	0.799245
	MR Egger	0.999671	0.997933-1.001412	0.713828	0.713828
	Weighted median	0.999831	0.999147-1.000515	0.628184	0.668293
	Weighted model	1.000327	0.999201-1.001455	0.573513	0.663006

The IVW-based MR estimates were further validated with other methods. However, the results of the MR Egger regression analyses showed two exceptions in the associations between AOSD and female NPs, as well as between rheumatoid arthritis and female NPs, where the corresponding FDR values were greater than 0.05 (FDR = 0.24072 for AOSD, FDR = 0.16095 for rheumatoid arthritis). The forest plots of the “leave-one-out” analyses were shown in the **Supplementary Materials** (**Table S4**). Furthermore, an insufficient number of SNPs were found when examining the effect of female NPs on the risk of AD traits (**Table S4**). The above results suggested causal effects of AOSD and rheumatoid arthritis on female NPs.

#### 3.1.2 Univariable MR results for male

**Figure 4:**
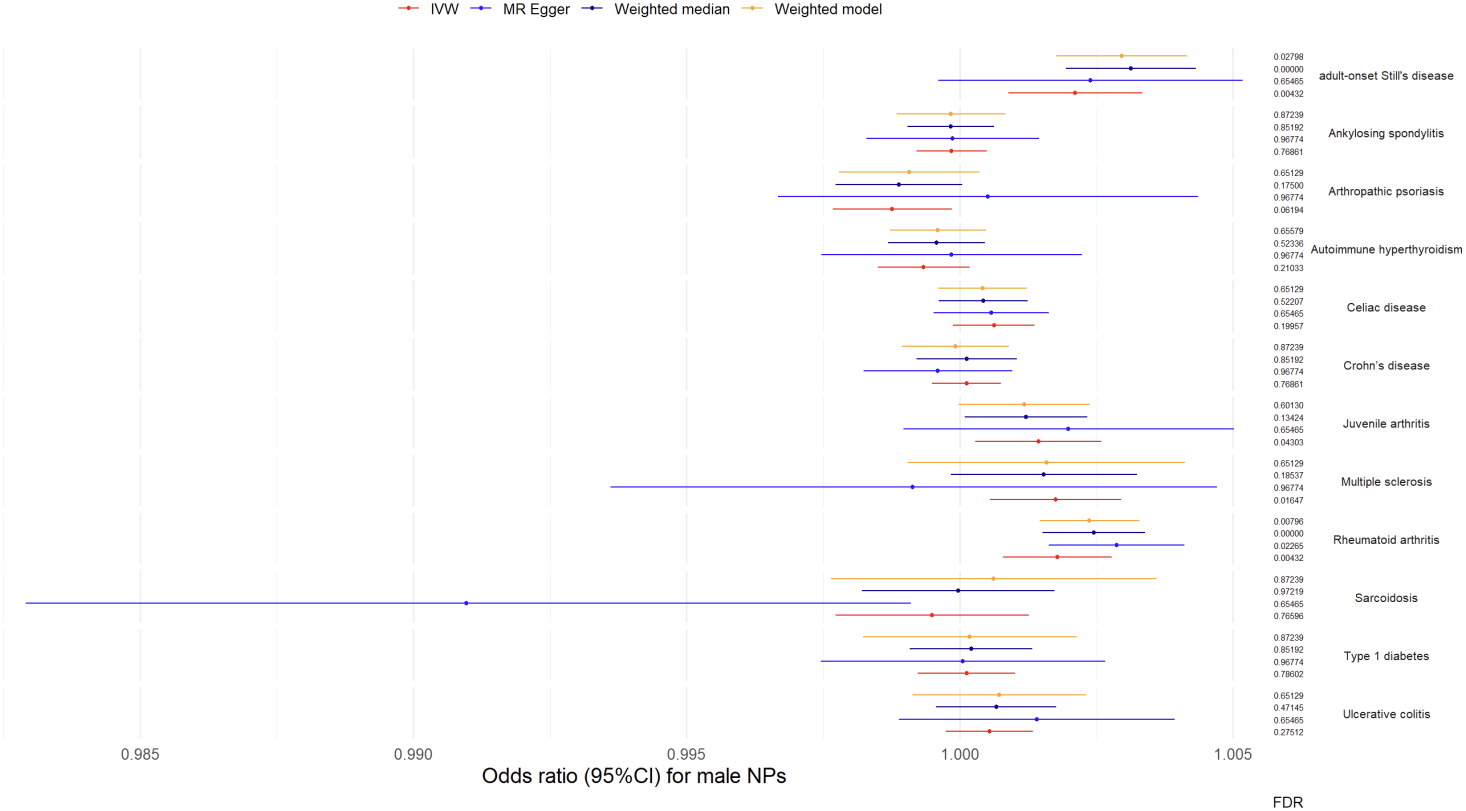
Odds ratio (95%*CI*) for male nasal polyps.

Results from univariable MR analyses showed causal associations between multiple ADs and NPs in males (**Figure 4**, **Table 2**). MR estimates from the IVW method suggested that 1-*SD* increase of inverse-normal transformed risk of AOSD (*OR* = 1.00211, 95% CI 1.000887-1.003336), rheumatoid arthritis (*OR* = 1.001784, 95% *CI* 1.000797–1.002772) and multiple sclerosis (*OR* = 1.001755, 95% *CI* 1.000556-1.002956) showed significant positive associations with male NPs risk, whereas 1-*SD* increase of inverse-normal transformed risk of ankylosing spondylitis (*OR* = 0.999848, 95% *CI* 0.999205–1.000491), autoimmune hyperthyroidism (*OR* = 0.999339, 95% *CI* 0.9985–1.000178), celiac disease (*OR* = 1.000623, 95% *CI* 0.999881–1.001365), Crohn’s disease (*OR* = 1.000122, 95% *CI* 0.999491–1.000754), sarcoidosis (*OR* = 0.999493, 95% *CI* 0.997725–1.001264), type 1 diabetes (*OR* = 1.000625, 95% *CI* 1.000098–1.001153), and ulcerative colitis (*OR* = 1.00054, 95% *CI* 0.999744–1.001337) were not significantly associated with male NPs risk. Of note, 1-*SD* increase of inverse-normal transformed risk of juvenile arthritis (*OR* = 1.001439, 95% *CI* 1.000287–1.002593) showed a suggestive positive association with male NPs risk, while 1-*SD* increase of inverse-normal transformed risk of arthropathic psoriasis (*OR* = 0.998762, 95% *CI* 0.997675–0.999850) showed a suggestive negative association with the male NPs risk.

**Table 2:** MR results for male.

traits	method	*OR*	95% *CI*	*p*	*FDR*
Adult-onset Still’s disease	IVW	1.00211	1.000887-1.003336	0.000721	0.004324
	MR Egger	1.002385	0.999601-1.005178	0.168536	0.654654
	Weighted median	1.00313	1.00194-1.004322	2.47E-07	1.67E-06
	Weighted model	1.002962	1.001765-1.004161	0.004664	0.027983
Ankylosing spondylitis	IVW	0.999848	0.999205-1.000491	0.643202	0.77502
	MR Egger	0.999868	0.998289-1.00145	0.874639	0.96774
	Weighted median	0.999837	0.999044-1.000632	0.688181	0.85902
	Weighted model	0.999838	0.998841-1.000836	0.758358	0.872386
Arthropathic psoriasis	IVW	0.998762	0.997675-0.99985	0.02581	0.070978
	MR Egger	1.000508	0.996671-1.00436	0.83867	0.96774
	Weighted median	0.998882	0.997726-1.000039	0.058334	0.212401
	Weighted model	0.999071	0.997787-1.000356	0.292074	0.68702
Autoimmune hyperthyroidism	IVW	0.999339	0.9985-1.000178	0.122694	0.224938
	MR Egger	0.999845	0.997466-1.002229	0.909936	0.96774
	Weighted median	0.999576	0.998688-1.000464	0.348909	0.548286
	Weighted model	0.999599	0.998722-1.000478	0.437194	0.68702
Celiac disease	IVW	1.000623	0.999881-1.001365	0.099785	0.219528
	MR Egger	1.000573	0.99952-1.001627	0.327327	0.72012
	Weighted median	1.000427	0.999612-1.001243	0.304543	0.548286
	Weighted model	1.000417	0.999603-1.001231	0.349341	0.68702
Crohn’s disease	IVW	1.000122	0.999491-1.000754	0.704563	0.77502
	MR Egger	0.999599	0.998234-1.000966	0.569069	0.96774
	Weighted median	1.00013	0.999212-1.001049	0.780927	0.85902
	Weighted model	0.999919	0.998937-1.000902	0.872386	0.872386
Juvenile arthritis	IVW	1.001439	1.000287-1.002593	0.014344	0.052596
	MR Egger	1.001988	0.998966-1.005019	0.326513	0.72012
	Weighted median	1.001212	1.000094-1.00233	0.033561	0.184583
	Weighted model	1.001176	0.999977-1.002376	0.150324	0.68702
Multiple sclerosis	IVW	1.001755	1.000556-1.002956	0.02264	0.02264
	MR Egger	0.999141	0.993608-1.004704	0.909936	0.96774
	Weighted median	1.001536	0.999832-1.003243	0.169921	0.212401
	Weighted model	1.001587	0.999055-1.004126	0.597018	0.68702
Rheumatoid arthritis	IVW	1.001784	1.000797-1.002772	0.000392	0.004309
	MR Egger	1.002869	1.001631-1.004109	0.001888	0.020763
	Weighted median	1.002455	1.001518-1.003393	2.79E-07	3.07E-06
	Weighted model	1.002374	1.001457-1.003291	0.000663	0.007294
Sarcoidosis	IVW	0.999493	0.997725-1.001264	0.574468	0.77502
	MR Egger	0.990973	0.982913-0.9991	0.117768	0.647726
	Weighted median	0.999969	0.998203-1.001738	0.972193	0.972193
	Weighted model	1.000615	0.997641-1.003597	0.706405	0.872386
Type 1 diabetes	IVW	1.000123	0.999232-1.001015	0.786018	0.786018
	MR Egger	1.000054	0.99746-1.00266	0.96774	0.96774
	Weighted median	1.000205	0.999087-1.001325	0.719385	0.85902
	Weighted model	1.000179	0.998225-1.002137	0.858786	0.872386
Ulcerative colitis	IVW	1.00054	0.999744-1.001337	0.183411	0.288217
	MR Egger	1.001408	0.998889-1.003934	0.284818	0.72012
	Weighted median	1.000666	0.999566-1.001767	0.235724	0.518592
	Weighted model	1.000723	0.999139-1.00231	0.379921	0.68702

The results of the IVW-based MR were largely consistent with those of the other methods. An exception could be found in the significant association between multiple sclerosis and male NPs, which was only supported by the IVW method. Therefore, the causal relationship between multiple sclerosis and male NPs was considered suggestive rather than conclusive. Besides, the *FDR* value of the MR Egger regression for AOSD was greater than 0.05 (*FDR* = 0.65465). The forest plots of the “leave-one-out” analyses were shown in the **Supplementary Materials** (**Table S5**). No potentially influential SNP was found for any of the exposure traits. Furthermore, no significant association was found when examining the causal effect of male NPs on the risk of autoimmune disease traits (**Table S5**). The above results suggest causal effects of AOSD and rheumatoid arthritis on male NPs.

### 3.2 MVMR results

**Figure 5:**
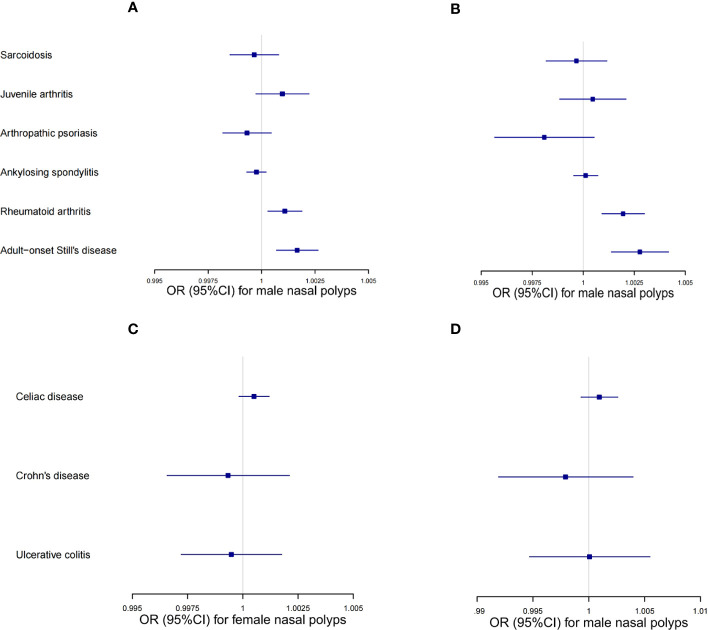
MVMR results of the connective tissue disease group and digestive system disease group. **(A)** MVMR odds ratio (95% *CI*) of the connective tissue diseases on female nasal polyps. **(B)** MVMR odds ratio (95% *CI*) of the connective tissue diseases on male nasal polyps. **(C)** MVMR odds ratio (95% *CI*) of the digestive system diseases on female nasal polyps. **(D)** MVMR odds ratio (95% *CI*) of the digestive system diseases on male nasal polyps.

During UMR analysis, we found shared IVs among several autoimmune diseases belonging to the connective tissue disease group, and a similar situation was also found in the digestive system disease group. As this may affect the reliability of UMR analysis, MVMR analyses were conducted to validate the associations between connective tissue diseases and NPs, as well as the associations between digestive system diseases and NPs, respectively. The effects estimated for AOSD on NPs were comparable to the univariable IVW estimate (*OR* = 1.001664, 95% *CI* 1.000697–1.002632 for females. *OR* = 1.002775, 95% *CI* 1.001379–1.004173 for males), while the effects estimated for rheumatoid arthritis on NPs were also comparable to the univariable IVW estimate (*OR* = 1.001086, 95% *CI* 1.000296–1.001876 for females; *OR* = 1.001952, 95% *CI* 1.000916–1.00299 for males). Other multivariable MR estimates for autoimmune diseases and nasal polyps were not significant (**Figure 5A-D**, **Table S6-9**). The MVMR results suggested that there were significant associations between ASOD and NPs, as well as between rheumatoid arthritis and NPs. Details are available on the website of “*Frontiers in Immunology*” (**Table S6-9**).

### 3.3 Mediation analysis

B-cell activating factor (BAFF), a member of the TNF superfamily, plays an important role in the generation, survival, proliferation, and maturation of B-cells (20, 21). Previous studies have shown that BAFF is upregulated in autoimmune diseases, as well as infiltrating in the submucosa of nasal polyps (22). Therefore, two-step MR analyses were conducted to investigate the mediating pathway from AOSD/rheumatoid arthritis to NPs via BAFF-related phenotypes. After screening in the IEU openGWAS database, we identified “BAFF-R on IgD+ B cell” as a mediator of the causal effect of rheumatoid arthritis on NPs. The mediation effect of 0.000126 (95% *CI*, 0.000054 to 0.000197; the mediated proportion of 7.06%) for female NPs, and the mediation effect of 0.000131 (95% *CI*, 0.000054 to 0.000207; the mediated proportion of 7.30%) for male NPs (**Table 3**). No mediator of AOSD on NPs was found.

**Table 3:** Mediation effect of BAFF-R on IgD+ B cell.

Mediator	Outcome	Indirect *β* (IVW 95%*CI*)	Indirect *se*	Mediated prop	indirect_*p*
BAFF-R on IgD+ B cell	female_NP	0.000126 (0.000054-0.000197)	0.000036	0.070592	0.042261
	male_NP	0.000131 (0.000054-0.000207)	0.000039	0.072966	0.048167

## 4 Discussion

Our study aimed to comprehensively investigate the potential causal relationship between ADs and NPs. To achieve this, we selected multiple ADs as exposure traits and stratified the outcome trait of nasal polyps by gender. After rigorous instrumental variable screening, we performed bidirectional univariable Mendelian randomization and multivariable Mendelian randomization analyses and found the causal effects of AOSD and rheumatoid arthritis on NPs. Through mediation analyses, we identified “BAFF-R on IgD+ B cell” as the mediator of the causal effects of rheumatoid arthritis on NPs. Besides, our study found that the risk of ankylosing spondylitis and celiac disease showed suggestive positive associations with female NPs, while the risk of juvenile arthritis and multiple sclerosis showed suggestive positive associations with male NPs. By contrast, the risk of arthropathic psoriasis showed a suggestive negative association with NPs. The study also found that sarcoidosis, ulcerative colitis, Crohn’s disease, type 1 diabetes, and autoimmune hyperthyroidism had no significant association with NPs.

There have been sporadic reports on the relationship between NPs and three of the twelve autoimmune diseases included in our study: autoimmune hyperthyroidism, ankylosing spondylitis, and rheumatoid arthritis. The case-control study of Choi et al. (23) found no significant association between autoimmune hyperthyroidism and nasal polyps, which is consistent with our findings. As mentioned in the introduction section, previous studies have shown conflicting results on the association between rheumatoid arthritis and NPs, as well as on the association between ankylosing spondylitis and NPs. Considering that Mendelian randomization methods have a higher level of evidence, our results make a constructive contribution to resolving these controversial issues. Moreover, we found a suggestive positive association between ankylosing spondylitis and female NPs but not male NPs, which partially explains the conflicting results of previous studies on the association between ankylosing spondylitis and NPs, as the association between ankylosing spondylitis and NPs risk is influenced by gender.

Shih et al. (5) have reported an association between rheumatoid arthritis and nasal polyps, which is consistent with our findings. Furthermore, our results provide evidence for a causal effect of rheumatoid arthritis on the risk of nasal polyps via “BAFF-R for IgD+ B cells”. BAFF-R is a specific receptor for BAFF, which is known to regulate signaling cascades that are crucial for the maturation and survival of IgD+ B cells (24, 25). Sokoya et al. (26) found that IgD plasma cells are prominent in sinus tissues and are increased in CRS. In addition, the level of IgD in the secretions of CRSwNP patients is higher than that of CRSsNP patients and the control group, indicating that activated IgD+ B cells are closely related to CRSwNP and supports our results. Besides, the study of Woo et al. (27) showed that compared with mild rheumatoid arthritis, severe rheumatoid arthritis has higher expression levels of NF-κB (p65 and p50), BAFF, and BAFF-R in the synovial membrane, while inhibition of NF-κB can reduce the expression of BAFF-R. This finding suggests that rheumatoid arthritis patients are likely to have upregulated BAFF-R due to the excessive activation of the NF-κB pathway. Exploring whether inhibiting the activation of the NF-κB signaling pathway in rheumatoid arthritis patients can reverse the development of CRSwNP is a worthwhile research direction in the future.

Additionally, our study found that the causal effects of ADs on male NPs are stronger than those on female NPs. This conclusion is based on several interesting facts. First, the odds ratio for the association between AOSD and male NPs is significantly higher than that for female NPs. This difference is observed in four different Mendelian randomization (MR) methods: inverse variance weighted (IVW), MR Egger regression, weighted median, and weighted model. The same situation is observed in the causal association between rheumatoid arthritis and NPs. Secondly, the results of the IVW analysis show that three ADs (AOSD, rheumatoid arthritis, and multiple sclerosis) have causal effects on male NPs, whereas only two ADs (AOSD and rheumatoid arthritis) have causal effects on female NPs. This gender bimodality may be related to differences in estradiol secretion levels. Espersen et al. (28) reported a 5-times greater odds (p = 0.01) of developing nasal polyposis in the presence of lowered estradiol plasma levels than in the presence of normal/elevated levels, suggesting that estradiol can inhibit the occurrence and development of nasal polyps and partially explains this phenomenon. Furthermore, Trivedi et al. (29) found that estradiol inhibits Group 2 innate lymphoid cells (ILC2s) function by suppressing NF-κB activation induced by IL-33. Considering that ILC2s are increasingly recognized as a key controller of type 2 inflammation and are well known to be highly elevated in type 2 inflammatory CRSwNP (30), this finding partly explains the specific mechanism of estradiol-induced sex bimodality. Further investigation of gender bimodality in the causal effects of ADs on NPs is required in the future.

To ensure the robustness of our results, we used reasonable approaches to select instrumental variables, conduct MR analyses, and perform sensitivity analyses. However, this study still has some limitations. First, the conclusions drawn from European populations may not apply to other populations. Second, the genetic characteristics of Finnish populations differ from those of other European populations, but this difference has not been taken into account in our study. Third, as this study aimed to obtain robust results, the suggestive significant causal effects of ankylosing spondylitis, celiac disease, and arthropathic psoriasis on female nasal polyps, as well as those of juvenile arthritis, multiple sclerosis, and arthropathic psoriasis on male nasal polyps, were not further investigated. Fourth, gender stratification of the exposure traits will further refine the experimental design and help produce more reliable results. However, since the sex-stratified data can only be obtained from the UKB database, and we have already used the UKB-derived data for the outcome trait, there would be a large overlap between the sample data of the exposure traits and that of the outcome traits if the exposure data were also derived from the UKB database. This would result in our study no longer meeting the basic requirements of a two-sample Mendelian randomization analysis, but more akin to a one-sample Mendelian randomization analysis. Considering the high false-positive rate and low reliability of single-sample randomization, it seems more feasible to stratify the outcome factor by sex only.

Overall, to the best of our knowledge, this is the study with the most autoimmune diseases included in research on the associations between autoimmune diseases and nasal polyps to date. For the first time, we investigated the causal relationships between nine autoimmune diseases and nasal polyps in different genders and found that: (1) there was a causal relationship between AOSD and nasal polyps; (2) sarcoidosis, ulcerative colitis, type 1 diabetes, and Crohn’s disease had no significant association with nasal polyps; (3) celiac disease showed a suggestive positive association with female nasal polyps, while juvenile arthritis and multiple sclerosis showed suggestive positive associations with male nasal polyps. By contrast, arthropathic psoriasis showed a suggestive negative association with nasal polyps. In addition to these nine diseases, previous controversial issues were further investigated: (1) there was a causal relationship between rheumatoid arthritis and nasal polyps, which was partially mediated by “BAFF-R for IgD+ B cells”; (2) ankylosing spondylitis showed suggestive positive associations with the female but not the male nasal polyps. We also confirmed that there was no causal effect of autoimmune hyperthyroidism on nasal polyps. By comparing the research results between different genders, we have initially observed the sex bimodality in the causal effects between autoimmune diseases and nasal polyps, with those on male nasal polyps being stronger than those on female nasal polyps. Our study lays a solid foundation for further research in the future, not only helping identify individuals susceptible to nasal polyps early but also improving our understanding of the immunopathogenesis of these heterogeneous diseases.

## Data availability statement

The original contributions presented in the study are included in the article/**Supplementary Material**. Further inquiries can be directed to the corresponding author.

## Ethics statement

All data were approved by the ethical review boards to which the original investigators belonged and each participant in the original study provided informed consent to the corresponding original investigators.

## Author contributions

SC conceived and designed the study, analyzed the data, prepared the figures, and drafted the paper. LT contributed significantly to data analysis and paper revision. DQ, HL, YYX, XW, and JH provided crucial assistance throughout the entire process. YX edited and revised the manuscript. All authors agree to be accountable for the content of this work. All authors contributed to the article and approved the submitted version.
